# Synergizing Algorithmic Design, Photoclick Chemistry and Multi‐Material Volumetric Printing for Accelerating Complex Shape Engineering

**DOI:** 10.1002/advs.202300912

**Published:** 2023-07-03

**Authors:** Parth Chansoria, Dominic Rütsche, Anny Wang, Hao Liu, Davide D'Angella, Riccardo Rizzo, Amelia Hasenauer, Patrick Weber, Wanwan Qiu, Nafeesah Bte Mohamed Ibrahim, Nina Korshunova, Xiao‐Hua Qin, Marcy Zenobi‐Wong

**Affiliations:** ^1^ Department of Health Sciences and Technology ETH Zürich University Zürich 8092 Switzerland; ^2^ Department of Surgery University Children's Hospital Basel 4056 Switzerland; ^3^ Hyperganic Group GmbH 80799 Munich Germany

**Keywords:** algorithmic design, auxetic, hydrogels, multi‐material, volumetric printing

## Abstract

The field of biomedical design and manufacturing has been rapidly evolving, with implants and grafts featuring complex 3D design constraints and materials distributions. By combining a new coding‐based design and modeling approach with high‐throughput volumetric printing, a new approach is demonstrated to transform the way complex shapes are designed and fabricated for biomedical applications. Here, an algorithmic voxel‐based approach is used that can rapidly generate a large design library of porous structures, auxetic meshes and cylinders, or perfusable constructs. By deploying finite cell modeling within the algorithmic design framework, large arrays of selected auxetic designs can be computationally modeled. Finally, the design schemes are used in conjunction with new approaches for multi‐material volumetric printing based on thiol‐ene photoclick chemistry to rapidly fabricate complex heterogeneous shapes. Collectively, the new design, modeling and fabrication techniques can be used toward a wide spectrum of products such as actuators, biomedical implants and grafts, or tissue and disease models.

## Graphical abstract

Accelerating the designing and manufacturing of complex shapes has been a driving factor of modern industrialization. Here, an algorithmic voxel‐based approach to rapidly generate porous structures, auxetic meshes and cylinders, or perfusable constructs is demonstrated. This design scheme in conjunction with new approaches for multi‐material volumetric printing to rapidly fabricate complex heterogeneous structures is used.

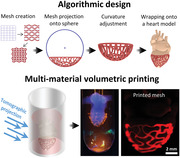

## 1 Introduction

Design complexities in the biomedical domain have been increasing at an exponential pace. This is highly relevant in the field of implants and grafts. Using advanced computer‐aided design (CAD) and computational modeling (CM) tools, complex implants are being developed that offer better mechanics (reduced weight and increased load‐bearing capability, etc.) or patient safety and comfort, as replacements of simpler implants. For example, the designs of arterial stents have transitioned from the conventional porous shapes to auxetic architectures,^[^ ^1^, ^2^ ^]^ which allow easy radial expansion of the stents while reducing the risk of stent malapposition and foreshortening.^[^ ^1^ ^]^ Such auxetic shapes have also recently paved the way to a new range of patches and tissue grafts for regenerative applications such as those repairing cardiac and pulmonary pathologies,^[^ ^3^, ^4^ ^]^ where auxetic patches allow easy conformation to organ deformation and outperform non‐auxetic patches.^[^ ^5^ ^]^ In addition, auxetic structures are increasingly being used as actuators or load‐bearing structures.^[^ ^6^, ^7^, ^8^, ^9^ ^]^ Furthermore, advanced CAD and CM tools are increasingly being used in tissue engineering to design complex perfusable structures, such as perfusable vascularized biomimetic tissue models for studying biogenesis and disease progression and treatment.^[^ ^10^, ^11^ ^]^ Unfortunately, to this date, CAD and CM still largely require manually defining the complex geometrical relationships and boundary conditions. Furthermore, one needs to analyze a wide array of design iterations to derive the optimized design for the application. For example, auxetic patches developed for different dynamic organs (lung, heart, etc.) need to conform to the stiffness and Poisson's ratios of the different organs, and may require analyzing hundreds of design iterations and their computational modeling to find the right patch design for an organ.^[^ ^4^ ^]^ Herein, we present a new algorithmic approach to designing complex shapes within seconds, which can rapidly generate a large array of design iterations within minutes.

To fabricate the complex shapes, additive manufacturing techniques involving layer‐by‐layer material deposition offer a wide range of achievable resolution (typically 10–500 µm) and throughput (0.01–1000 mm^3^ hr). Recently, volumetric printing (VP), also termed as volumetric additive manufacturing, has emerged as a powerful technique toward the fabrication of high‐resolution structures (up to 100 µm) within tens of seconds. VP relies on computed axial lithography, where the vial containing photocrosslinkable resin (photoresin) is rotated with dynamically evolving light patterns (images) projected into the resin.^[^ ^12^, ^13^, ^14^ ^]^ The superposition of the projected images leads to a spatially localized increase in the free radicals produced from the photoinitiator, which induces cross‐linking of the photoresin into the desired shape. The photo‐rheology of the resins used in VP typically depicts a non‐linear response, where the cross‐linking is induced after a threshold of light dose is achieved. There are also non‐rotational methods involving image projections from multiple sides (typically front, side and bottom) to generate a spatially localized increase in light dose within the photocrosslinkable matrix, which induces cross‐linking of the material into the desired shape.^[^ ^15^, ^16^ ^]^ Compared to multi‐direction projections, computed tomography leads to better resolution and shape fidelity of the printed construct, and allows fabrication of more complicated bespoke architectures, as the image can be changed continuously.^[^ ^12^ ^]^ We recently demonstrated that the tomographic printing duration can be further reduced to only a few seconds by using thiol‐ene photoclick chemistry‐based resins.^[^ ^17^ ^]^ Herein, insensitivity to oxygen and a homogeneous network formation within the step‐growth polymerized matrices can reduce internal structural stresses and shrinkage after printing compared to constructs resulting from chain‐growth polymerization.^[^ ^17^, ^18^ ^]^ Further, refractive index (RI) matching and fine‐tuning of light dose has enabled high resolution printing while allowing encapsulation of high density of cells and organoids,^[^ ^19^, ^20^ ^]^ which are critical to biomimetic tissue engineering. To set a roadmap for high throughput design and fabrication of biomedical implants and grafts, we demonstrate how complex algorithmically designed structures can be rapidly synthesized using photoclickable matrices within VP.

**Figure 1:**
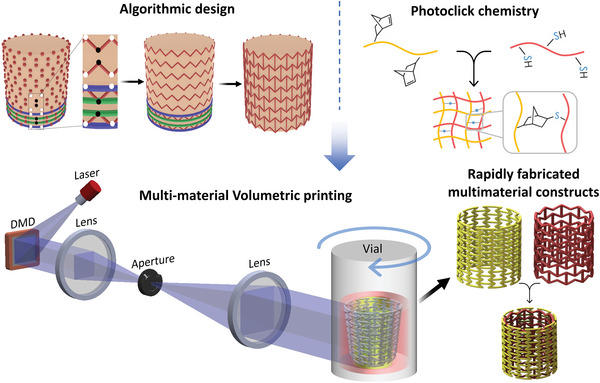
Schematic of the proposed concept to rapidly design and fabricate complex structures. Algorithmic design is used to rapidly create large arrays of design iterations, photoclick chemistry‐based bioresins allow rapid fabrication of constructs, and multi‐material volumetric printing approach rapidly fabricates complex constructs made from heterogenous resin compositions.

Furthermore, applications of VP have been mostly limited to constructs based on single material compositions. Notably, one of the first works on tomographic light projection for printing demonstrated the concept of having another pre‐fabricated solid component (they showed printing of a screwdriver head) integrated within the print container during the tomographic printing.^[^ ^12^ ^]^ Recently, melt electrowritten scaffolds have been embedded within the print chamber to fabricate fabrication of tubular constructs with enhanced mechanical properties. Unfortunately, incorporation of more than one volumetrically printable material during the tomographic projection process has not yet been explored. New methods to fabricate complex multi‐material structures can widen the applicability of VP to biomimetic structures as well as tissue and disease models. In this work, we demonstrate new approaches for multi‐material VP. We print selected architectures with different photoresin compositions along the length or the thickness of the constructs. Further we demonstrate how the designs can be wrapped around more complex objects such as a heart, which paves the way for rapidly printing organ‐specific auxetic meshes. Finally, we also highlight how complex multi‐material perfusable architectures such as alveoli can be rapidly designed and fabricated using our approach. The synergy of algorithmic design, photoclick chemistry and multi‐material VP (**Figure** **1**) offers a transformational approach to rapidly designing and fabricating complex multi‐material shapes.

## 2 Results

### 2.1 Rapid Algorithmic Design and Volumetric Printing of Auxetic Meshes

**Figure 2:**
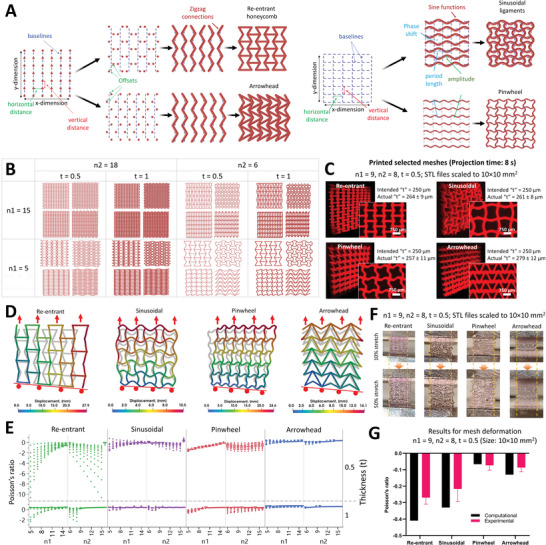
Rapid design and fabrication of auxetic meshes. A). Design rationale for creating different auxetic meshes – Re‐entrant honeycomb, arrowhead, sinusoidal ligaments, and pinwheel (see Supporting Information for governing equations). B). Rapidly generated design iterations for the auxetic architectures by varying select design parameters (n1, n2 and t represent the number of vertical and horizontal elements, and thickness of the elements, respectively). C). Light sheet microscopy images of selected volumetrically printed meshes (design parameters n1, n2 and t are listed in the figure), and the corresponding micrographs. Actual thickness of the printed elements closely correlates with the intended thickness. D). Algorithmically derived computational estimates of structural mechanics of selected architectures (original mesh is shown in grey), demonstrating the negative Poisson's ratios of the structures (i.e., the structures expand laterally when stretched longitudinally). In these results, a roller constraint was applied on the bottom of the mesh to allow free lateral expansion. E). Computational estimates of the Poisson's ratios of the different auxetic meshes (244 designs were analyzed per auxetic mesh; computation time 4.5 s/design), based on different combinations of n1, n2 and t. Of note: The thickness has been plotted as a second y axis on the right. F). Selected meshes being expanded from a 10% pre‐strain to a 50% strain in a tensile testing apparatus. Width of the expanded meshes is quantified from the captured images, and Poisson's ratio is computed. G). Experimentally derived Poisson's ratios closely correspond to the computational estimates.

Auxetic shapes serve as an ideal template for algorithmic design as the structural interrelationships can be defined via governing equations. 2D auxetic meshes have been increasingly utilized as patches that feature tailorable negative Poisson's ratios and directional stiffness to easily conform to dynamic organs such as the lung or the heart.^[^ ^3^, ^4^, ^22^ ^]^ **Figure** **2**A illustrates the algorithmic design scheme for auxetic meshes featuring re‐entrant honeycomb, sinusoidal ligaments, arrowhead and pinwheel meshes. All of these meshes feature different stiffness and Poisson's ratios, which can be selectively matched to different dynamic organs (e.g., lung, heart, stomach, bladder, etc.).^[^ ^4^, ^5^ ^]^ The equations governing the design algorithms have been provided in the Supporting Information. Briefly, for re‐entrant honeycomb meshes, vertical baselines containing the vertices of the mesh elements are established at a defined separation, followed by offsetting the position of every alternate vertex either ahead (positive offset) or behind (negative offset) the baseline. Herein, every consecutive baseline has opposing offsetting of the vertices (i.e., if the vertex at any baseline has a positive offset from the vertex, then the consecutive baseline will have a negative offset from the vertex). Next, the vertices are connected along the vertical direction, followed by connecting alternate vertices that are at positive and negative offset, along the horizontal direction. For the arrowhead architectures, the meshes are offset such that each baseline features the same offset pattern of the vertices. The vertices are then connected such that the negative offset vertices of any baseline are connected to the positive offset vertices along the consecutive baseline. For creating the sinusoidal ligament meshes, sine functions are created with period length (2*π*) spanning two baselines, and the sinusoidal mesh in every other baseline is shifted by a phase spanning the distance between two consecutive baselines. The pinwheel meshes are fabricated in a similar way, except there is no offset between the sinusoids across the baselines. In Figure 2B, we present select architectures created by changing dimensions of the auxetic design features. Using this algorithmic approach, several hundred iterations of any auxetic mesh can be rapidly created (average compilation time per design on a personal computer at 1.3 GHz and 32 GB RAM was 0.04 s). Figure 2C demonstrates selected volumetrically printed meshes made using Rhodamine (Rhod)‐labeled fluorescent resin containing norbornene‐modified gelatin (GelNB) and thiolated gelatin (GelSH) at 5% w/v (total gelatin content) in phosphate buffered saline (PBS) (see dose optimization and resolution tests for different photoclick materials in Figure S1, Supporting Information). The use of photoclick thiol‐ene reaction within allowed us to rapidly print (≈8 s of tomographic projection time per mesh) 10×10 mm^2^ meshes at a high resolution and fidelity (thickness of the mesh elements in Figure 2C closely correlated with the intend design values).

### 2.2 Algorithmically Defined Computational Models for Rapidly Screening Structural Mechanics

Similar to the algorithmic design schemes, we show that algorithmic computational modeling can also substantially accelerate the process of determining the mechanics of a wide array of complex structures. Here we use the auxetic meshes as the templates for our computational models based on the finite cell method. As opposed to the conventional approaches, the embedded simulation approach eliminates the necessity of the labor‐intensive procedure for meshing and defining of boundary constraints. Instead, the geometry is embedded in a regular grid with vanishing stiffness. The physical model is then recovered by applying a voxel‐based integration rule.^[^ ^24^ ^]^ Using this methodology allowed us to computationally model the auxetic meshes and cylinders at an average duration of ≈4.5 s in a 1.3 GHz personal computer with 32 GB RAM. As a result, we were able to quickly derive the Poisson's ratios of thousands of auxetic structures without any manual intervention. The deformation of selected meshes have been shown in Figure 2D, and the collective computational outcomes of the Poisson's ratios of the meshes are shown in Figure 2E. Of these, the re‐entrant meshes demonstrate the widest range of Poisson's ratios (from 0.1 to −10.9) based on the different combinations of the design features (n1, n2 and t). Such a wide range of Poisson's ratios for the different meshes and cylinders offers unique applications such as in actuators,^[^ ^6^, ^7^ ^]^ structural components,^[^ ^8^, ^9^ ^]^ implants,^[^ ^25^, ^26^ ^]^ or organ‐specific patches.^[^ ^3^, ^4^ ^]^ We also validated the computational estimates within a tensile testing apparatus (Figure 2F) using the selected volumetrically printed meshes (Figure 2C). Here, comparing the width of the constructs under different axial strains allowed us to determine the Poisson's ratios (‐ΔW/ΔL, where W and L are the width and length of the meshes, respectively). The experimental outcomes of the Poisson's ratios closely correlated with the computational outcomes (Figure 2G) for the different geometries, but were, on average, smaller in magnitude compared to the computational outcomes. Deviation between the computational and experimental outcomes could be attributed to the differences in boundary constraints between the computational (roller constraint) and experimental (fixed constraint) setups. A fixed constraint in the experimental setup, where the mesh extremities were sandwiched in between metal jaws, prevented free lateral expansion of the mesh, which may result in the mesh exhibiting lower Poisson's ratios. Another reason could be that the hydrogel‐based design elements within the meshes tended to stick to each other when the experiments were performed in air (Figure 2F), which may have prevented them from opening‐up when the meshes were stretched. In future, we could perform the stretching of the meshes under water or use a balloon model to further characterize the auxetic characteristics of the meshes,^[^ ^4^, ^27^ ^]^ to validate the computational models.

### 2.3 Multi‐Material Volumetric Printing of Auxetic Meshes

**Figure 3:**
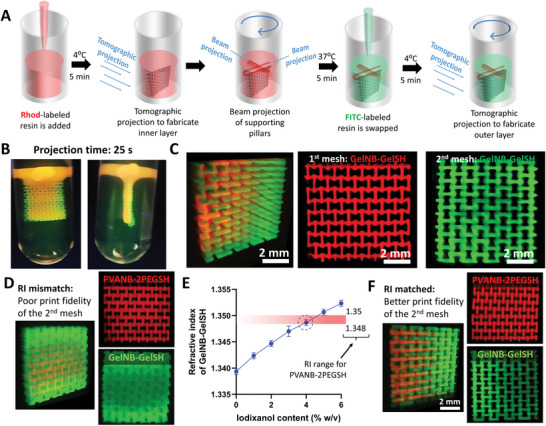
Fabrication of auxetic meshes featuring different resin compositions across the thickness of the meshes. A). Supports projection and resin‐swapping scheme of fabrication of the multi‐material auxetic meshes featuring different designs and resin compositions along the thickness. B). Rapidly fabricated auxetic meshes (25 s total tomographic projection time) featuring horizontally and vertically oriented re‐entrant auxetic meshes made of Rhodamine (Rhod)‐labeled, FITC‐labeled GelNB/GelSH resins, respectively, along the thickness. C). Images of the meshes captured using light sheet microscopy. D). Meshes printed using Rhod‐labeled PVANB‐2PEGSH, followed by printing of FITC‐labeled GelNB‐GelSH, where the GelNB‐GelSH meshes features a poor print resolution. E). RI of GelNB‐GelSH resin is matched to that of PVANB‐2PEGSH by addition of Iodixanol, where 4% w/v of iodixanol allows matching of the RI range for the PVANB‐2PEGSH resin. F). Shape fidelity of the GelNB‐GelSH constructs is improved after RI matching.

With auxetic meshes as the template, we present the first scheme for the rapid multi‐material (**Figure** **3**A). Here, the inner mesh is created using tomographic projections in a vial filled with thermo‐reversibly crosslinked Rhod‐labeled GelNB‐GelSH. After the first tomographic projection, the mesh is localized in the resin container by projecting supporting beams (created by projecting a circular image (Φ = 3 mm) for 5 s without rotating the vial) at the top of the mesh. This prevents the printed structure from falling when the vial is heated up to 37 °C to remove the non‐photocrosslinked resin. The resin in the container is then interchanged with a FITC (fluorescein isothiocyanate)‐labeled GelNB‐GelSH resin, followed by tomographic projections of the outer mesh. Addition of either Rhodamine or FITC did not affect the absorption of light at 405 nm and the RI of the resin (RI = 1.34 for GelNB/GelSH), thereby not affecting the light path during tomographic projections. Figure 3B demonstrates the rapidly fabricated (25 s total tomographic projection time) bi‐layered auxetic mesh comprising of different resin compositions (Rhod‐labeled and FITC‐labeled GelNB/GelSH) and vertically and horizontally oriented re‐entrant honeycomb meshes in the first and second layers, respectively. The images in Figure 3C have been captured using light sheet microscopy,^[^ ^28^ ^]^ and the supporting pillars facilitate image capturing by stabilizing the constructs within the printing vials. Notably, post photocrosslinking, the RI of the GelNB/GelSH resin increases by ≈0.002 (i.e., new RI = 1.342), which, as per our observations, did not critically affect the print fidelity of the second mesh. The minimum feature size of the second mesh (≈289 µm) was within ±10% as that of the first mesh (≈264 µm). Next, within the same printing scheme (Figure 3A) we used a different the first mesh to norbornene‐modified polyvinyl alcohol (PVANB) containing 2‐arm thiolated PEG (PVANB‐2PEGSH) based on our previous work,^[^ ^29^ ^]^ which features a higher RI range (1.348–1.35) compared to GelNB‐GelSH (1.339–1.34). Here, we observed a poor print fidelity of the second mesh, which could be attributed to the enhanced scattering effects due to the presence of a cross‐linked construct in the resin that has a substantially different RI.^[^ ^19^ ^]^ Here, we matched the refractive index of the GelNB‐GelSH resin by adding iodixanol to the resin, which is a biocompatible RI matching agent, previously used in the context of cell‐laden resin, to increase the RI of the resin to match that of the cells (1.375–1.38).^[^ ^19^ ^]^ We identified that 4% w/v of Iodixanol increases the RI of the GelNB‐GelSH resin to match that of the PVANB‐2PEGSH resin. This minimized the scattering effects due to the previously printed PVANB‐2PEGSH‐based mesh and allowed printing of constructs at a higher shape fidelity. Of note, porcine gelatin was added at 3% w/v concentration to the PVANB‐2PEGSH resin to allow the thermo‐reversible gelation needed for successful volumetric printing. In a previous work, we have shown that this sacrificial gelatin can slowly diffuse out of the resin under incubation.^[^ ^29^ ^]^

**Figure 4:**
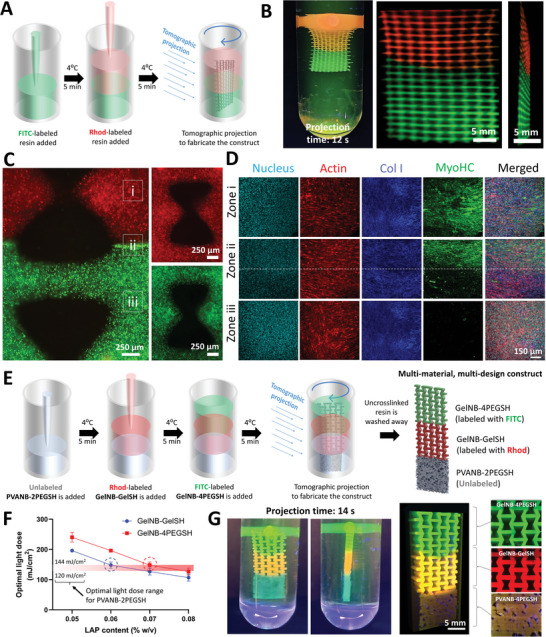
Fabrication of constructs featuring different resin compositions across the length. A). Scheme of filling different resin compositions and printing the entire construct at once. B). Printed constructs featuring Rhod‐labeled or FITC‐labeled GelNB/GelSH matrix across the two layers. C). Micrographs of muscle‐connective tissue model made of GelNB/GelSH resin. The top and bottom portions comprise of C2C12 myoblasts labeled with cell tracker red and 3T3 fibroblasts labeled with cell tracker green, respectively. D). After 4‐week long culture in DMEM containing 2% w/v horse serum, the muscle‐mimicking mesh containing C2C12 cells (Zone i) exhibits Myosin heavy chain (MyoHC) staining. The interface at Zone ii shows the distinction in MyoHC staining across the two layers, whereas the connective tissue‐mimicking mesh containing 3T3 cells (Zone iii) does not demonstrate MyoHC staining. E). Scheme of printing a three‐layered construct featuring PVANB‐2PEGSH, GelNB‐GelSH and GelNB‐4PEGSH with different geometries across different regions. F). LAP  content in the GelNB‐GelSH and GelNB‐4PEGSH resins was increased to match that of PVANB‐2PEGSH, such that the entire construct with the three different resins can be printed at once using a single tomographic projection. G). Camera images of the printed constructs (supporting pillars are added to better visualize the constructs), and light sheet microscopy images demonstrating the three different regions of the constructs, each featuring a different material composition.

Of note, the supporting pillars in the first multi‐material VP scheme need to be removed by post printing. The second printing scheme for multi‐material VP does not require projection of supporting pillars. This scheme is demonstrated in **Figure** **4**A. The scheme utilizes filling the first resin in the printing vial, followed by adding the second resin. Here, the two resin compositions are prevented from mixing into each other through thermo‐reversible cross‐linking of each resin formulation at 4 °C prior to adding the subsequent one. Alternatively, a high viscosity resin could also be used to prevent mixing of the resins during the short printing duration. After the different resins are added, the entire construct is printed at once via tomographic projections (tomographic projection time = 12 s). We use this multi‐material VP technique to fabricate a re‐entrant honeycomb mesh with vertically oriented elements featuring FITC and Rhod‐labeled GelNB/GelSH resin in the bottom and top portions, respectively (Figure 4B). Of note, the supporting beams are still added to the construct to suspend it within the printing vial, which facilitates its imaging using light sheet microscopy. Here, gelatin‐based resin has been used since it allows the layers to thermo‐reversibly cross‐link when the temperature is reduced, thereby preventing the photocrosslinked structures from falling under their own weight due to gravity. The gelatin could also be replaced with other materials such as pluronic or hyaluronic acid, etc., provided they increase the viscosity substantially to prevent the structures from displacing during the short printing duration. This multi‐material VP approach can be used to print tissue interfaces. As an example, we printed the same auxetic mesh with GelNB/GelSH across the top and bottom, but the top compartment consisted of myoblasts (C2C12 murine), and the bottom compartment consisted of fibroblasts (3T3 murine), labelled with cell tracker red and green, respectively (Figure 4C). After 4 weeks of maturation, the top compartment selectively exhibited myo‐heavy chain staining, while both compartments featured collagen I stain (Figure 4D). In future, we plan to maturate the constructs for longer time periods and use more compliant and bioactive matrices (such as those featuring collagen or Matrigel),^[^ ^30^ ^]^ so the myotubes could demonstrate spontaneous contractility.

While we only demonstrated two resin compositions within the previously printed constructs, the technique can be adapted to more than two material compositions to create more complex constructs. To demonstrate such an example without cells, we created three‐material constructs featuring a porous matrix (based on a foaming algorithm; outcomes in Figure S2, and further details in the Supporting Information) at the bottom (Figure 4E), and auxetic lattices for the middle and top regions. For this, we printed with an unlabeled PVANB‐2PEGSH resin, Rhod‐labeled GelNB‐GelSH resin in the middle region, and FITC‐labeled GelNB‐4PEGSH (i.e., GelNB with 4‐arm thiolated PEG as the cross‐linker, based on our previous work)^[^ ^17^ ^]^ in the top region. Notably, the three resins required different light doses (Figure S1, Supporting Information). Therefore, we kept the PVANB‐2PEGSH resin as the template, as it required the lowest light dose for printing, and increased the photoinitiator ( Lithium phenyl(2,4,6‐trimethylbenzoyl)phosphinate; LAP) concentration within the other two resins to be able to match the light dose range for PVANB‐2PEGSH (120–144 mJ cm^−2^). As increasing the LAP concentration reduces the required light dose for printing, a LAP concentration of 0.06% and 0.07% w/v was found to be adequate for the GelNB‐GelSH and GelNB‐4PEGSH resins, respectively (Figure 4F). Next, for preparation of the printing vial, we sequentially added each resin in the vial and performed thermoreversible gelation for 5 min at 4 °C prior to adding the consecutive resin. Matching the light doses for the different resins allowed us to print the three‐material construct using a single tomographic projection image sequence; printed construct is shown in Figure 4G.

In this scheme of multi‐material VP, the print file was carefully positioned in the software of the volumetric printer (Tomolite, Readily3D), such that the different print regions feature different materials and designs. Despite precise positioning, one concern that arises here is the formation of the meniscus due to surface tension of the resin with the walls of the print vial, where the interface is not across a plane (i.e., a straight line when viewed from the side; evident in both Figure 4B,G). In future, we plan to avoid this by tuning the design at the interfaces that accounts for the curvature due to surface tension. In future, we also plan to use this technique to print using cell‐laden models, to create tissue interfaces such as a bone‐tendon‐muscle interface. Here, considerations for hybridizing VP with other processes such as FLight or extrusion printing can allow us to achieve more biomimetic arrangement such as an interspersed fibers in the myotendinous junction.^[^ ^31^, ^32^ ^]^ This has been further elaborated in the discussion section.

### 2.4 Rapid Algorithmic Design and Multi‐Material Volumetric Printing of Auxetic Cylinders

**Figure 5:**
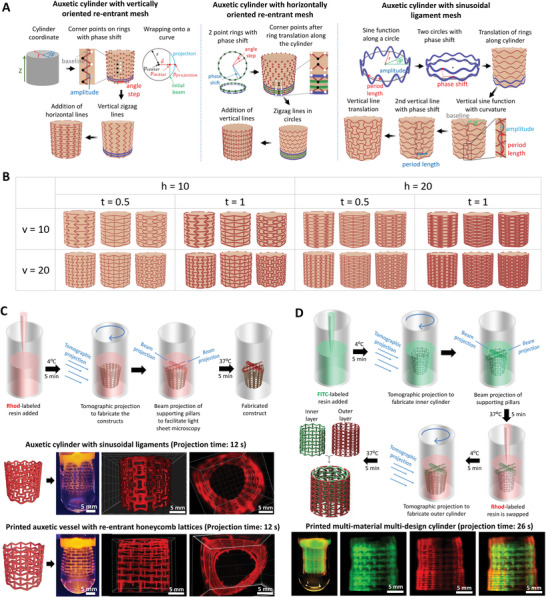
Rapid design and fabrication of auxetic cylindrical meshes. A). Algorithmic design rationales for the fabrication of auxetic cylindrical constructs featuring re‐entrant and sinusoidal ligament meshes (see Supporting Information for governing equations). B). Design arrays featuring selected iterations of the design parameters (h, t, and v represent the number of horizontal lines from top to bottom, thickness of the elements, number of vertical lines in the ring, respectively). C). Rapid fabrication of select architectures using volumetric printing, and compiled micrographs of the printed architectures (images are captured using the light sheet microscopy). D). Resin swapping scheme for the fabrication of bi‐layered cylindrical auxetic meshes and printed meshes demonstrating horizontally and vertically oriented re‐entrant honeycomb architectures across the inner and outer layers, respectively.

Cylindrical auxetic meshes, such as those increasingly being used for fabrication of stents can be even more complicated to design when compared to 2D meshes,^[^ ^1^, ^2^ ^]^ especially when the design elements need to form a continuum across a cylindrical contour. Here, creating design iterations of the auxetic cylinders is particularly challenging. **Figure** **5**A illustrates the algorithmic design scheme of cylindrical auxetic meshes featuring re‐entrant honeycomb and sinusoidal ligament elements (detailed equations have been provided in the Supporting Information). The design schemes of auxetic cylinders featuring arrowhead or pinwheel architectures have been provided in the Figure S3 (Supporting Information). For creating any auxetic mesh, we first convert the cartesian coordinate system to a cylindrical coordinate system to be able to create a cylinder and define points on it. For auxetic cylinders featuring vertically oriented re‐entrant honeycomb lattices, we create vertical zigzag lines by defining the coordinates of all corner points alternating on two sides of a base line (Figure 5A). The alternate corner points lie along parallel circles with a phase shift commensurate with the width of the re‐entrant meshes. After all the corner points of the vertical zigzag lines are defined, these relate to straight beams. To match the cylinder curvature, every point on the initial straight beam is translated onto the cylinder using a wrapping algorithm (details provided in the Supporting Information). Finally, to create the horizontal beams, every second pair of neighboring points on each ring of the cylinder is connected by an arc using the same wrapping method, with the positions of the horizontal beams alternating along the axis of the cylinder. For auxetic cylinders featuring horizontally oriented re‐entrant honeycomb meshes, the corner points of each zigzag ring are composed of two circles consisting of points with a certain phase shift and angular step to each other. After defining all corner points of the zigzag rings, these can be connected. Before generating the connecting beams, every beam between two corner points is translated onto the cylinder coat using the wrapping method used previously. Finally, vertical beams are constructed between neighboring zigzag rings. For the auxetic cylinders with sinusoidal ligament meshes, sinusoidal functions are created along the cylinder, with consecutive functions featuring a phase shift with the previous sinusoid. Next, a vertical sine wave is generated to create the auxetic cylinders with sinusoidal elements. The starting and ending points of a vertical line are on the crossing points of the bottom and top circles and their baselines respectively. Similar to the 2D auxetic meshes, this algorithmic design scheme allows us to create a wide array of cylinders within a matter of seconds (Figure 5B; Figure S3, Supporting Information). Furthermore, we can also use the algorithmic computational schemes to determine the structural mechanics of the cylinders along both the axial and the radial directions (Figure S4, Supporting Information). In comparison to the auxetic meshes (Figure 2), the auxetic cylinders (selected outputs are shown in Figure S4A, Supporting Information) demonstrate negative Poisson's ratios in the ranges of 0.2 to −2.4, with both Pinwheel and Re‐entrant honeycomb architectures offering large range of Poisson's ratios (Figure S4B, Supporting Information). Interestingly, most designs of the Pinwheel architectures do not demonstrate a unform radial expansion when the meshes are stretched axially, which can be attributed to the twisting of the constitutive elements that is compounded across the length of the constructs. Interestingly, the trends for the dependencies of the Poisson's ratios based on the different design elements (n1, n2, h, v, and t) are similar between the meshes and the cylinders featuring the same design elements. Unlike the axial stretch, none of the designs feature negative Poisson's ratios when stretched radially, but a wide range of positive Poisson's ratios. This means that the cylinders are contracting when they are stretched radially, even though the meshes feature auxetic designs. Here, the cylinders featuring horizontally oriented re‐entrant honeycomb elements demonstrate Poisson's ratios varying from 0.2 to 1.1 based on different combinations of the design features (h, v, and t). We plan to experimentally validate the computational models of the cylindrical meshes and develop functional architectures, as a part of our future studies.

These auxetic architectures can also be printed within 12 s using photoclick resins within VP (Figure 5C). Using the first multi‐material VP strategy of using support pillars to allow the construct to be suspended in the printing vial, which allows changing of the resin and re‐printing, we also demonstrate how multi‐material and multi‐design auxetic cylindrical meshes can be fabricated. Figure 5D demonstrates auxetic cylindrical meshes made of FITC‐labeled GelNB/GelSH in the shape of horizontally oriented re‐entrant honeycomb mesh in the inner layer, and Rhod‐labeled GelNB/GelSH in the shape of vertically oriented re‐entrant honeycomb mesh in the outer layer, respectively.

### 2.5 Organ‐Specific Auxetic Meshes

**Figure 6:**
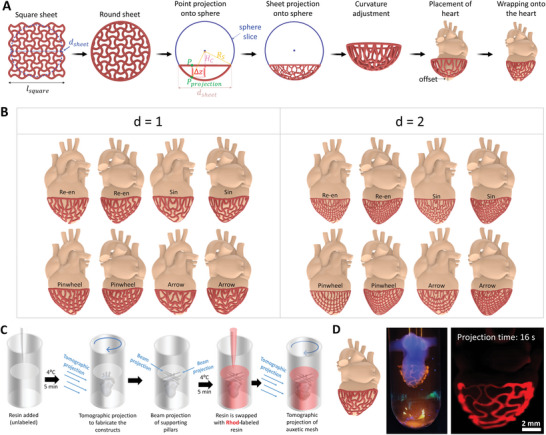
Creation of organ‐specific auxetic meshes using wrapping algorithms. A). Rationale for the fabrication of the curved auxetic meshes and wrapping the same onto custom organ models (in this case, a heart). B). Design iterations of different auxetic meshes wrapped around the heart (d represents the density index of the auxetic structure. C). Resin swapping procedure for the fabrication of the heart construct with a wrapped auxetic mesh. D). Macroscopic image (left) and light sheet microscopy image (right) of the heart construct (non‐labeled) with auxetic mesh (Rhod‐labeled) around it.

To demonstrate the level of design complexity that the algorithmic schemes can address, we demonstrate how the auxetic meshes can be wrapped around more complex shapes such as the heart (**Figure** **6**A). Here, we start with a square 2D auxetic mesh, which is intersected with a circle to derive a circular mesh. Then, all points of this circular mesh are wrapped onto a sphere (Figure S5, Supporting Information). The formation of a curved mesh is an essential step, as it facilitates the wrapping algorithm to identify the points within the mesh closest to the surface of the heart models. After the curved is constructed, the heart model (in this case, a standard tessellation language (STL) file derived from an online repository^[^ ^33^ ^]^) is placed into the curved auxetic sheet such that they intersect slightly with each other, which improves the wrapping result in the next step. Finally, every point on the auxetic sheet is projected onto the bottom of the heart by computing the point on the heart surface with the smallest distance to a given point on the curved sheet. As with previous shapes, all the different kinds of auxetic meshes can be wrapped onto the heart this way (average design compilation time ≈0.2 s) (Figure 6B, also see Figure S6, Supporting Information for additional shape iterations). The corresponding governing equations for the design schemes are provided in the Supporting Information, and other simpler shapes such as spheres are shown in Figure S5 (Supporting Information). Here, we select the sinusoidal meshes to demonstrate their rapid printability over a heart model (Figure 6C). For this, we first volumetrically print a heart using unlabeled GelNB/GelSH resin, followed by projecting supporting pillars and swapping the non‐photocrosslinked resin with Rhod‐labeled GelNB/GelSH. The auxetic mesh around the heart is then rapidly volumetrically printed Figure 6D. Notably, the auxetic meshes in Figure 6D were printed directly over the heart to allow better visualization of the mesh by maintaining its shape. We foresee that such complex shapes may one‐day pave the way for patient‐specific grafts or patches that can conform to the shape of the organ.

### 2.6 Algorithmic Design of Perfusable Architectures

**Figure 7:**
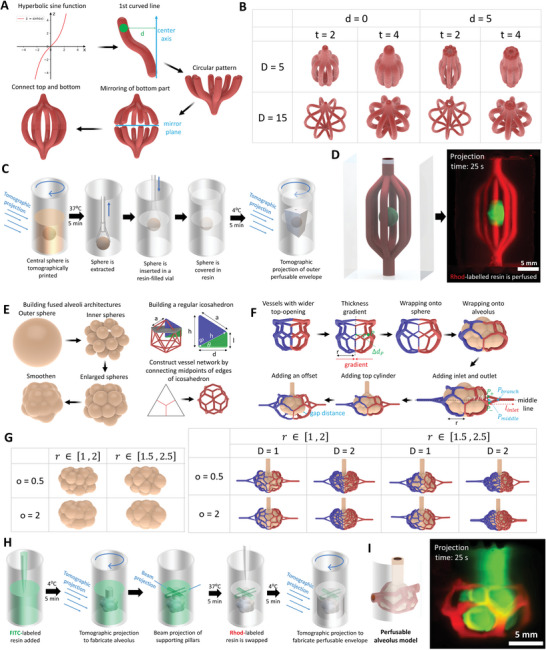
Rapid design and fabrication of perfusable constructs. A). Design rationale for simple perfusable architectures featuring multiple bifurcating channels. B). Select architectures made under iterations of design parameters (d, t and D represent inlet channel diameter, thickness of individual channels and diameter of the offset of the channels at the center). C). multi‐material VP scheme, where the central FITC‐labeled sphere (GelNB/GelSH) is created first, and then transferred to another resin container pre‐filled with unlabeled resin (GelNB/GelSH) and tomographic projections are performed. D). Volumetrically printed construct after perfusion with Rhod‐labeled resin (images through light sheet microscopy). E). Scheme of fabrication of the alveolar budding structures and the perfusable channels. F). Wrapping of the perfusable channels around the alveolus. G). Shape iterations of alveolar structures (r represents radius of individual mini‐spheres and o represents the offset of spheres w.r.t. each other) and the perfusable channels (D represents the density of capillaries) around the alveoli. H). Scheme of fabrication of the alveolar construct (also see Figure S7, Supporting Information). I). Printed construct with FITC‐labeled alveolus construct with perfused Rhod‐labeled resin surrounding the construct (images captured through light sheet microscopy).

Finally, we demonstrate how the algorithmic design and multi‐material VP schemes can be used to design and fabricate simple and complex perfusable structures. In the first design scheme, we design a cuboid consisting of a hollow spheroidal center and six perfusable channels around the sphere. The channels are created as a hyperbolic sine function and a circular pattern is created by copying the sine functions 7 times around the center axis (**Figure** **7**A). Next, the channels are mirrored with an offset in the middle, followed by connecting the mirrored and original parts through straight channels. By changing the amplitude of the hyperbolic sine function or the diameter of the channels, a variety of perfusable shapes can be created within seconds (Figure 7B). These channels are then removed from a cuboidal shape. In addition, a sphere with a pre‐set offset with the channels is also removed from the cuboid to result in the perfusable construct with a hollow sphere in‐between. To fabricate a construct featuring perfusable channels surrounding a matrix of different material, we introduce the third multi‐material VP approach – prefabricated construct integration (Figure 7C). In this approach, a FITC‐labeled GelNB/GelSH sphere is fabricated, followed by extracting the same and integrating within another resin container partially filled with unlabeled GelNB/GelSH resin. The resin is kept at 24 °C to allow easy integration of the sphere. More unlabeled GelNB/GelSH resin is then filled over the construct, and the cuboidal construct with hollow sphere and perfusable channels printed such that the prefabricated spherical construct is accommodated within the hollow spherical center in the construct. Perfusing Rhod‐labeled GelNB/GelSH into the channels allows us to image the sample using light sheet microscopy, as shown in Figure 7D, where the central FITC‐labeled sphere is surrounded by a network of Rhod‐labeled channels. We foresee that such synergistic algorithmic design and multi‐material VP approach can find potential applications in disease‐on‐a‐chip models, for instance, a tumor surrounded by a network of capillaries that demonstrate neovascularization to the tumor site. Such a model can be used to study the effects of biological (macrophages,^[^ ^34^ ^]^ exosomes,^[^ ^35^ ^]^ etc.) or non‐biological therapeutics (e.g., chemotherapies) on tumor metastasis or angiogenesis.^[^ ^36^, ^37^ ^]^

The algorithmic design framework is not just limited to simple perfusable shapes but can also be expanded to more complex shapes such as alveoli surrounded with a perfusable vessel network.^[^ ^38^ ^]^ The alveolus is created by dividing a sphere into several smaller spheres bound by the periphery of the sphere, followed by scaling‐up individual spheres to create the budding alveolus structure (Figure 7E). The vessel network surrounding the alveolus is first created as a regular icosahedron (see governing equations in the Supporting Information). Next, every equilateral triangle along the face of the icosahedron is divided into four identical equilateral triangles by connecting the midpoints of all edges. This process can be repeated to further divide the triangles and create denser structures with smaller hexagons. Then, for every equilateral triangle, its geometric center relates to the midpoint of each edge to create the vessel network (Figure 7E). Further, to improve the quality of prints and avoid intersection with the top cylinder later, the vessel network on the top is enlarged by factor 2 to create a bigger opening (Figure 7F). A thickness gradient is introduced to the vessels, such that the thickness decreases gradually from the left and right ends of the shape towards the middle. Then every edge of the icosahedron is interpolated to create 11 equidistant intermediate points, i.e., 10 sub‐beams per edge, followed by wrapping each onto the circumscribed sphere of the icosahedron. The result is a spherical vessel shape that can be used for wrapping. The alveolus is placed such that it is concentric with the vessel shape, and every beam on the vessel shape is interpolated into 10 sub‐beams and wrapped onto the alveolus by computing the point on the alveolar surface with the smallest distance to a given point on the vessel network. Then a cylinder is created and placed on top of the alveolus, and inlet and outlet ports added such that the inlet/outlet port intersects with a branching point of the vessel network. In the present case, we created the vessels at an offset by using a larger alveolar shape for wrapping of the vessels, then placing a smaller alveolar shape concentrically for the final shape. By changing the offset, vessel diameters and their densities, we could generate a wide array of alveoli and surrounding vessel shapes (average design compilation time ≈0.5 s) as shown in Figure 7G. In the model we used for printing, we used a gap of 250 µm between the alveolus and the vessels. The alveoli and the vessel network were removed from a cylindrical construct to create perfusable channels and a hollow portion to accommodate the alveolus. The alveolus construct for printing was also hollowed‐out by removing a scaled‐down shape from the original alveolus. The printing scheme utilized first printing the alveolar shape, using FITC‐labeled GelNB/GelSH, followed by supporting pillar projection (Figure 7H). This was followed by swapping resin with unlabeled GelNB/GelSH and printing the hollow construct (see the printed constructs during different stages in Figure S7, Supporting Information). Finally, uncross‐linked Rhod‐labeled GelNB/GelSH at 37 °C is perfused in the channels (Figure 7I), and the entire construct exposed to 405 nm UV light to cross‐link the resin in the channels to facilitate light sheet microscopy. In our future work, we plan to print different vessel densities followed by seeding of epithelial cells to create lung‐on‐chip models, which can be used to study pulmonary pathologies.^[^ ^39^ ^]^

## 3 Discussion

The algorithmic design scheme offers a transformational approach toward facile creation of a wide array of complex shapes, such as the auxetic meshes and cylinders, organ‐specific grafts or perfusable alveoli structures that we demonstrated in this work. The algorithms can rapidly process a wide array of point‐point connections (such as wrapping functions, segmentation of icosahedron triangles into smaller triangles, connective lattice elements of auxetic shapes, etc.) that would be tedious and time‐consuming to execute manually. Once the design scheme is established for any shape, the shapes can be easily iterated by inputting the ranges and increments for the important parameters, which is otherwise a daunting task to perform in conventional CAD software. This difference in designing and iterating is even more profound as the shape complexity increases, with the introduction of organ‐specific auxetic meshes and the alveolar structures. Here the algorithms ensure that the interconnections between the constitutive points or contours are satisfied when a new shape iteration is formed. Notably, previous work on printing of functional multivascular networks^[^ ^40^ ^]^ had demonstrated the use of mathematical space‐filling and fractal topology algorithms for their structural design. However, the use of multiple software frameworks including Blender and Solidworks for designing the curves, vascular networks, and intravascular designs, may collectively render the designs difficult to alter. In contrast, a single algorithmic design framework allows us to render thousands of design iterations within minutes.

We have also shown that algorithm‐based computational modeling schemes can allow rapid screening of the mechanical properties of large arrays of complex architectures. Here, integration of the iterative algorithmic design and computational modeling within a deep learning framework, such as that described by Wilt et al.,^[^ ^41^ ^]^ can lead to a powerful framework for shape optimization for specific applications. Notably, while work by Wilt et al.^[^ ^41^ ^]^ work was constrained to 2D planar or cylindrical shapes, it has the potential to be tuned to more complicated applications that we have demonstrated, such as the meshes wrapped around organs (e.g., to provide mechanical support to the myocardium after an infarction),^[^ ^42^ ^]^ or the perfusable networks (e.g., to allow optimal nutrient diffusion).^[^ ^40^ ^]^ Our future work will entail expanding the computational modeling to accommodate organ deformation, and also simulate fluid flow within perfusable architectures. In the content of developing prosthetic implants,^[^ ^26^ ^]^ future studies could also investigate development of an algorithmic design and computational framework to design and distribute different auxetic elements in a metamaterial implant, and then analyze the structural mechanics of the implant to be able to identify the biomedically relevant implant design.

We have made the design and simulation code available in the GitHub repository (see “**Data and materials availability**” section). For users who are not adept at coding, we have created graphical user interfaces where the users can load their own models, to be able to design their own custom auxetic patches or perfusable networks with varying vessel densities. Using the interface, the users can also iterate the designs of the auxetic and perfusable shapes demonstrated in the present work for their own applications. In addition, dedicated libraries have been established that can be used for executing the structural simulations or for creating porosities within constructs.

In this work, we deployed three techniques for the rapid volumetric printing of multi‐material constructs: 1). Tomographic projection printing of the first layer of the construct supported by projected pillars, followed by swapping of the uncross‐linked resin with a different composition and tomographic projection printing of the subsequent layer. 2). Filling the printing vials with two different resin compositions and tomographic projection printing of the entire construct at once. 3). Incorporating a prefabricated construct into a resin‐filled container, followed by tomographic projections to fabricate the multi‐material constructs. The rationale for which multi‐material VP method needs to be used would depend on the complexity of the structure that needs to be fabricated, and the resin composition. While the first scheme offers tremendous design freedom (we printed multi‐material auxetic cylinders (Figure 5) auxetic mesh on heart (Figure 6) or perfusable alveolar models (Figure 7) using this method), aligning subsequent projections with the first projection is often challenging. Furthermore, removal of the supporting beams after printing can lead to material losses and may even be difficult to execute for fragile constructs. For multi‐material constructs such as tissue interfaces, the second scheme can be a more robust scheme to align the two layers (such as the bilayered auxetic meshes in Figure 3). However, for resin compositions that do not undergo thermo‐reversible cross‐linking, the second scheme may cause mixing of the two resins across different regions, especially when the resin viscosity is low. Here, the use of sacrificial gelatin with appropriate RI matching can be a prudent choice, or the remaining two strategies could be used. Notably, the change in RI before and after cross‐linking of GelNB/GelSH resin in most of the prints we have shown was small (ΔRI = 0.002). Therefore, we did not observe a substantial difference in resolution between different stages of tomographic projections. However, if the base material is different between the projections, the differences in RI between the photocrosslinked constructs can cause unwanted scattering or diffraction of light that may affect the print resolution and quality, as we demonstrated in the example where the PVANB‐2PEGSH‐based construct was first printed, and then the resin was swapped to print the GelNB‐GelSH‐based construct (Figure 3). Similar constraints may also apply to the third strategy of prefabricated construct integration. In this case, RI matching agents such as Iodixanol can be used to fine‐tune the RI of the resins and achieve high resolution prints.^[^ ^19^ ^]^

To alleviate all of these concerns with different materials being printed, one could, in fact, print with a single resin composition where the constitutive materials can cross‐link under different wavelengths.^[^ ^43^, ^44^ ^]^ This way, multi‐material constructs could be printed without having to replace the resins between different prints, or adding multiple resins within the print chamber. For instance, Wang et al.^[^ ^44^ ^]^ used a single material capable of being volumetrically printed multiple wavelengths (365 and 455 nm) in the context of stiffness control within the constructs. Such a technique could be adapted to cross‐link different material constituents within a single resin by using different wavelengths for tomographic projection, where the non‐cross‐linked constituents can simply diffuse away after printing. Here, dose optimization for printing of different constituents, and the effect of multiple photoinitiator species on cells, may be non‐trivial aspects to characterize.

While we have demonstrated VP in the current work, hybridization of VP with other processes may allow the fabrication of functional structures. For instance, in a scheme of hybridization of VP with FLight,^[^ ^32^ ^]^ one could use FLight to fabricate the muscle and tendon interfaces with fascicular arrangement of muscle fibers or aligned collagen in tendons, while the bone can be tomographically projected to create a porous matrix. In fact, hybridization can also be performed with other printing techniques.^[^ ^45^, ^46^ ^]^ For example, extrusion printing of photoresins can be used to control the spatial distribution of different materials within the resin container. Subsequently, single tomographic projection image sequence can be used to create the multi‐material constructs. Here, dose matching for the different material may be necessary, as we have demonstrated in Figure 4F. Such hybridization schemes will be the future scope of our investigation. Naturally, for tissue engineering purposes, imparting a macroscopic vasculature, and further inducing neovascularization will also be a key aspect of our future research to allow physiological‐scale tissue fabrication.^[^ ^10^, ^47^ ^]^

For this work, in order to demonstrate rapid printing, photoclick materials based on step‐growth polymerization were ideal as we have established expertise on high speed volumetric printing using these materials.^[^ ^17^ ^]^ For most of the prints shown in the present work, we simply used the GelNB‐GelSH resin as it demonstrated a good print resolution (≈200 µm, Figure S1, Supporting Information), and that GelSH was more easily available (we could synthesize it in the lab) and affordable than 4PEGSH or 2PEGSH (procured commercially). However, it is important to note that the use of cross‐linkers featuring more tightly controlled molecular weight distribution and degree of substitution, such as the 2‐arm or 4‐arm PEGSH, could reduce batch‐to‐batch variability and improve the predictability of matrix properties. Furthermore, the materials demonstrated in this work may not be suitable toward all biomedical or structural applications, as the modulus is small (≈10–100 kPa). Herein, one could also use chain‐growth polymerization, which is typically found in acrylate or methacrylate‐based resins, to obtain stiffer structures. Future research on double network hydrogels,^[^ ^48^ ^]^ of which one material is based on click chemistry can yield higher stiffness constructs and could improve the applicability of the materials to a wider variety of biomedical applications such as polymer‐based arterial stents or tracheal grafts.^[^ ^49^, ^50^ ^]^ Further, current volumetric printing approaches have been limited to constructs spanning only a few centimeters in sizes, and future research on tomographic projections within larger containers can circumvent such size limitations. As such, one also does not need to use volumetric printing or deploy photocrosslinkable materials. The shapes generated and optimized through the algorithmic design and computational modeling schemes can be fabricated though conventional or bespoke manufacturing processes integrated into larger assembly lines, if the complexity of the shape can be achieved. For example, the algorithmic schemes could help speed up product design and optimization of prosthetic implants or metallic stents, to even vehicle drive shafts and engines, which could bring about substantial cost‐savings in the product pipeline. This work can potentially transform the way engineers or scientists approach new design problems and develop solutions that have the potential to benefit society at large.

## 4 Experimental Section

### Algorithmic Design

Hyperganic core (an algorithmic engineering platform developed by Hyperganic GmbH) environment was used to run the algorithmic design schemes written in C#. Detailed explanations and equations of the algorithms were provided in the Supporting Information. We have created graphical user interfaces within Hyperganic core that will allow users to change the designs of the auxetic and perfusable shapes. User Interfaces had been created for each design architecture with provision to create designs based on variations of design parameters (h, t, v, n1, n2, etc.), and were integrated in the Hyperganic source code shaped on the open‐source library. See “Data and materials availability” section for the source codes for the design schemes and procedures for opening the graphical user interfaces to change different designs.

### Computational Modeling of the Structural Mechanics of the Auxetic Meshes

Numerical analysis of auxetic meshes had been performed using the simulation kernel of Hyperganic Core (governing equations for the models were provided in the Supporting Information). The simulation functionality was integrated within the C# API that directly integrates with the algorithmic design schemes. See “Data and materials availability” section for the sources codes and procedures for running the codes.

### Matrix Synthesis

The norbornene or thiol‐modified gelatin were synthesized using procedures previously established in the lab.^[^ ^17^, ^51^ ^]^ For formulation of GelNB, porcine‐derived (Type A) gelatin was dissolved in 0.5 m carbonate‐bicarbonate buffer (pH∼9, obtained by adding 38.2 g L^−1^ of sodium bicarbonate and 4.7 d L^−1^ of sodium carbonate in deionized (DI) water) at 50 °C to get a 10% w/v solution. After obtaining a clear solution under stirring, carbic anhydride was added at a concentration of 100 mg g^−1^ of gelatin. After letting the reaction proceed for 1 h, the solution was dialyzed (at 40 °C) with frequent DI water changes (2 per day) for 5 days. The matrix was then lyophilized for 4 days and stored at −20 °C until further use. For formulation of GelSH, porcine‐derived (Type A) gelatin was dissolved in 0.15 m MES (2‐(N‐morpholino)ethanesulfonic acid) buffer (pH∼4) at 50 °C to get a 2% w/v solution. When completely dissolved, DTPHY (3,3′‐Dithiobis(propionohydrazide)) was added at 95 mg g^−1^ of gelatin while stirring. When completely dissolved, EDC (1‐Ethyl‐3‐(3‐dimethylaminopropyl)carbodiimide) was added at 135 mg g^−1^ of gelatin while stirring. The reaction was then allowed to proceed at 50 °C under stirring for 12 h. Next, TCEP was added at 344 mg g^−1^ of gelatin, followed by continuing the reduction reaction for 6 h. Finally, 1 g of NaCl was added and the solution dialysed against MilliQ water balanced to pH 4.5 with diluted HCl. The degree of functionalization of the matrices was determined using ^1^H NMR spectroscopy (GelSH DS: 0.276 ± 0.016 mmol g^−1^, GelNB DS: 0.217 ± 0.007 mmol g^−1^, plots provided in Figure S8, Supporting Information).

PVANB was formulated and characterized as per the previous studies.^[^ ^29^, ^52^ ^]^ Briefly, in a three‐neck flask, 7.28 g PVA (47 kDa, Sigma–Aldrich no. 10 853) was dissolved in 120 mL anhydrous DMSO at 60 °C under argon protection, and then ≈10% v/v of the solvent was removed by distillation under vacuum. Afterward, 15.2 mg p‐toluenesulfonic acid was dissolved in 1 mL anhydrous DMSO and added into the PVA solution. Cis‐5‐Norbornene‐endo‐2,3‐dicarboxylic anhydride (5.21 g, abcr GmbH) dissolved in anhydrous DMSO (10 mL) was added dropwise to the mixture. After a 24 h reaction at 55 °C, the solution was cooled down to room temperature and transferred into a dialysis tube (Mw cut‐off: 6–8 kDa). After dialysis against NaHCO_3_ solution (1 m, pH 8.0) for 24 h and then against MilliQ water, the dialysate was lyophilized. The degree of functionalization was 13% as determined by 1H NMR analysis in D2O (Figure S9, Supporting Information). The PVANB‐2PEGSH resin was constituted by adding 2.5% w/v PEGNB, 2.5% w/v 2PEGSH (2 kDa molecular weight, Advanced BioChemicals), 3% sacrificial gelatin and 0.05% w/v LAP in PBS.

The GelNB/GelSH resin was formulated by mixing the lyophilized GelNB or GelSH matrix in PBS to achieve 5% w/v total gelatin concentration. GelNB/PEGSH resin was formulated by mixing the lyophilized GelNB matrix in PBS at 3.8% w/v and thiolated 4‐arm PEG (10 kDa, SinoPEG) at 1.2% w/v. For both resin formulations, 0.05% w/v LAP was used as the photoinitiator. Rhod‐ or FITC‐labeled resin was formulated by adding Acryloxyethyl thiocarbamoyl Rhodamine B (Rhod‐Acr) or Fluorescein isothiocyanate (FITC) stock in DMSO (at 10 mg mL^−1^) to the resin formulation at 1 µl mL^−1^. FITC was conjugated to the resin matrix through amide bond formation with the primary amines of the matrix, while Rhod‐Acr conjugates to GelSH through thiol‐ene reaction during printing. The amounts of FITC and Rhod‐Acr do not affect the light dose of the resin compared to a non‐labeled resin.

### Multi‐Material Volumetric Printing

The open format volumetric printer from Readily3D was used for these experiments. This printer allowed for each resin swapping and visualization of the constructs during and after printing. Procedures for multi‐material VP had been discussed in the results section. Here it was provide necessary details for replicability. Prior to printing, dose tests were performed by projecting an array of circles (ɸ = 1 mm) featuring a variation of light intensity onto a 3 mm path length cuvette filled with the resin. Diameters of the projected cylinders were then measured using bright field microscopy and the light dose allowing for the diameter to be 1 ± 0.05 mm (dimensions measured in ImageJ) was chosen. For all prints, 18 mm printing vials were used. After filling‐in the desired volume of the resin – 4 mL per layer for single material VPs and 3 mL per material for the multi‐material VP experiments, the resin was allowed to thermo‐reversibly crosslink for 5 min at 4 °C. This allowed stability of the printed structure during photocrosslinking. To remove the non‐cross‐linked resin (to swap the resin in between different tomographic projections or at the end of the printing), the vial was kept at 37 °C for 5 min, followed by washing with warm (37 °C) PBS twice. The supporting pillars were fabricated in a sequential manner: First a single circular beam projection at highest laser power permissible by the printing system (64 mW cm^−2^) was used for 10 s to print the first pillar. The vial was then rotated at 90°, and the projection performed again.

### Cell Culture and Tissue Immunohistochemistry

C2C12 murine myoblasts and NIH 3T3 murine fibroblasts were cultured in Dulbecco's Modified Eagle Medium (DMEM) supplemented with 10% w/v fetal bovine serum (FBS) and 1% w/v penicillin/streptomycin. The cells were passaged at 80% confluency using 0.25% w/v trypsin and 0.05% w/v EDTA. Next, C2C12 and 3T3 cells were labeled with CellTracker^TM^ red and green dyes (ThermoFisher), respectively, using manufacturer's specifications. The cells were then resuspended in GelNB/GelSH matrix at 2.5 m cells mL and the muscle‐connective tissue units volumetrically printed. After 4 weeks of culture, muscle‐connective tissue units were immunohistochemically stained for myosin heavy chain (MyoHC), Collagen I, Actin filament (Phalloidin) and nuclei (DAPI) based on the previous work.^[^ ^32^ ^]^

### Light Sheet Microscopy

An axially scanned light sheet microscope (MesoSPIM, V4) was used to image fluorescently labelled samples.^[^ ^53^ ^]^ The constructs were mounted onto a custom printed microscope sample holder and submerged in a quartz cuvette filled with mQ water, which was then mounted onto the MesoSPIM microscope stand. For imaging, a macro‐zoom system (Olympus MVX‐10) and 1x air objective (Olympus MVPLAPO1x) with adjustable zoom were used. Voltage adjustments using the electrically tunable lens (ETL) were performed for each run. Step size was chosen from 10–50 µm.

## Conflict of Interest

The authors declare no conflict of interest.

## Author Contributions

P.C. and M.Z.W. performed conceptualization. P.C., D.R., A.W., R.R., H.L., P.W., D.D., W.Q., X.Q., N.K., M.Z.W. performed methodology. P.C., D.R., A.W., R.R., H.L., W.Q., X.Q., P.W., N.K. performed investigation. P.C., D.R., A.W., and N.K. performed visualization. M.Z.W., P.C., and N.K. supervised the study. P.C., D.R., and A.W. wrote the original draft. M.Z.W., and P.C. wrote, reviewed, and edited.

## Supporting information

Supporting Information

## Data Availability

The data that support the findings of this study are openly available in ETH Research Collection at https://doi.org/10.3929/ethz‐b‐000583621, reference number 583621.
